# Use of an ultra-clean air flow for surgical field asepsis when performing intravitreous injections in an ambulatory surgical environment

**DOI:** 10.1186/s40942-020-00258-5

**Published:** 2020-11-19

**Authors:** Renata Moreto, Francyne Veiga Cyrino, Rodrigo Jorge

**Affiliations:** Ophthalmology, Ribeirão Preto Medical School, University of São Paulo, Avenida Bandeirantes, 3900, Ribeirão Preto, SP 14049-990 Brazil

**Keywords:** Endophthalmitis, Surgical infection, Intravitreous injections, Asepsis techniques, Ultra-clean air flow

## Abstract

**Background:**

Intravitreal injection of medications is one of the most common procedures performed in ophthalmology. Intravitreal anti-VEGF agents are currently the chosen treatment for ocular fundus diseases, including age-related macular degeneration and diabetic retinopathy. As an invasive procedure it involves risks. The most serious complication from intravitreal injection of anti-VEGF agents is endophthalmitis (EO). Although rare, EO can result in devastating loss of vision. This article evaluates whether the use of an ultra-clean air flow (UA) can be another useful tool in the prevention of EOs. Accordingly, the maintenance of asepsis of the surgical field of intravitreal injections was verified with and without the use of UA.

**Methods:**

The study was conducted in operating room of an ambulatory surgery center on four different surgical days when just intravitreal injections were scheduled. Two experiments using two Blood Agar and two Chocolate Agar plates (first 2 days; 4 plates by day) were carried out by positioning an UA directed to the surgical table and two other experiments (last 2 days; 4 plates per day) were carried out using similar plates without the use of the UA. All Blood Agar and four Chocolate Agar plates were positioned on the surgical table, close to the surgical filed. At the end of the day, after the conclusion of the intravitreous injections, the plates were sent for a biomolecular study that was carried out after 1 day of incubation at 37 °C.

**Results:**

The sixteen plates, eight Blood Agar and eight Chocolate Agar, were analyzed qualitatively for the growth or not of microorganism’s colonies and identification of their species. The biomolecular study demonstrated the growth of bacteria of the genus *Micrococcus* sp. with the use of the UA and without the the UA bacterias of the *genera Bacillus sp, Staphylococcus haemolyticus, Staphylococcus aureus* and *Staphylococcus cohnii ssp urealyticus* were found.

**Conclusion:**

The use of UA close to the operating table prevented the growth of pathogenic bacteria and should be considered as an alternative tool to avoid the contamination of materials and drugs used for intravitreal injections.

## Background

The development of targeted molecular therapy to inhibit vascular endothelial growth factor (VEGF) has revolutionized the treatment and visual prognosis of retinal diseases such as diabetic retinopathy and age-related macular degeneration, as well as macular edema and retinal vein occlusion through performing intravitreal injections (IVIs) of these drugs [1, 2]. As an invasive procedure it involves risks. The most serious complication from IVIs of anti-VEGF agents is endophthalmitis (EO). Despite the incidence of EO after anti-VEGF IVI is very low (0.038 to 0.065%) [3, 4], it may result in partial visual loss or even blindness [5, 6].

Etiological agents that usually cause EO are fairly abundant in the conjunctival flora of the normal human eye. The most usual ones are coagulase-negative staphylococci (CONS), most commonly *Staphylococcus epidermidis* followed by *Streptococcus viridans,* which is especially related to contamination of the operating room when there is frequent conversation during the IVIs procedures [7]. *Staphylococcus aureus, Bacillus* spss and *Pseudomonas* spss are also important microorganisms to be considered in the etiology of EO [2, 8].

In Canada and in USA, IVIs are mainly performed in the office [9], whereas in other countries IVI are limited to the operation rooms (OR) or to a sterile room with hygienic standards in order to decrease the risk of infection and EO. Important hygienic practices include hand hygiene, appropriate OR clothing, face masks for physicians and staff assisting in IVIs, the use of sterilized surgical fields, and isolation of the eyelashes and topical administration of 5% povidone-iodine 30 s before IVIs [9–11]. In addition, methods that may improve the quality of the OR air, such as ultraclean room ventilation, should be considered [13, 15]. In this context, in the present study, we evaluated the use of a new ultra-clean air flow (UA) as a possible tool to prevent contamination of the operating table. The ultra-clean airflow was obtained by means of a mobile air filter (Operio Mobile^®^, Toul Meditech), which produces a directed, non-turbulent ultra-clean airflow over the sterile instruments. The airflow speed is 0.4–0.5 m/s and has a capacity to clean the air of 400 m^3^/h and a protection area up to 120 cm. This system uses a HEPA filter that eliminates particles smaller than 0.3 µm from the air [14], and, consequently, may contribute to avoid contamination of the surgical filed and EO.

## Methodology

The study was conducted in the OR of the Ophthalmology sector at Hospital das Clínicas of the University Hospital, School of Medicine of Ribeirão Preto, University of São Paulo, a tertiary health care hospital.

The study was carried out on four different surgical days. The analyzes were performed as follows: the surgical tables were always positioned in the same place. On each day of IVIs, two Blood Agar and two Chocolate Agar were placed in the same location, at a distance of 120 cm from the site where the UA system would be located and in the same place without the UA. Two days, 11/29/2018 and 12/6/2018 with the UA directed to the operating table. Two days, 12/12/2018 and 12/19/2018, without the use of the UA. A total of 16 culture plates were used. In total 99 IVIs were done with the UA and 102 without it.

**Fig. 1:**
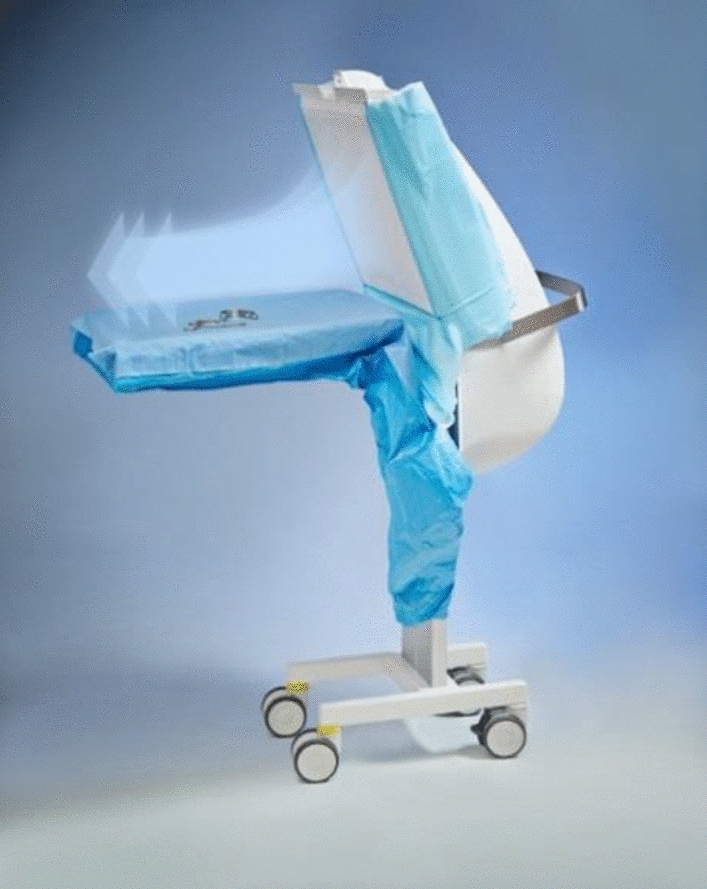
Operio Mobile^®^ equipment with a HEPA filter. The arrows represent the direction of the airflow. Image obtained from www.toulmeditech.com/en/products/6 at 30/03/2019)

**Fig. 2:**
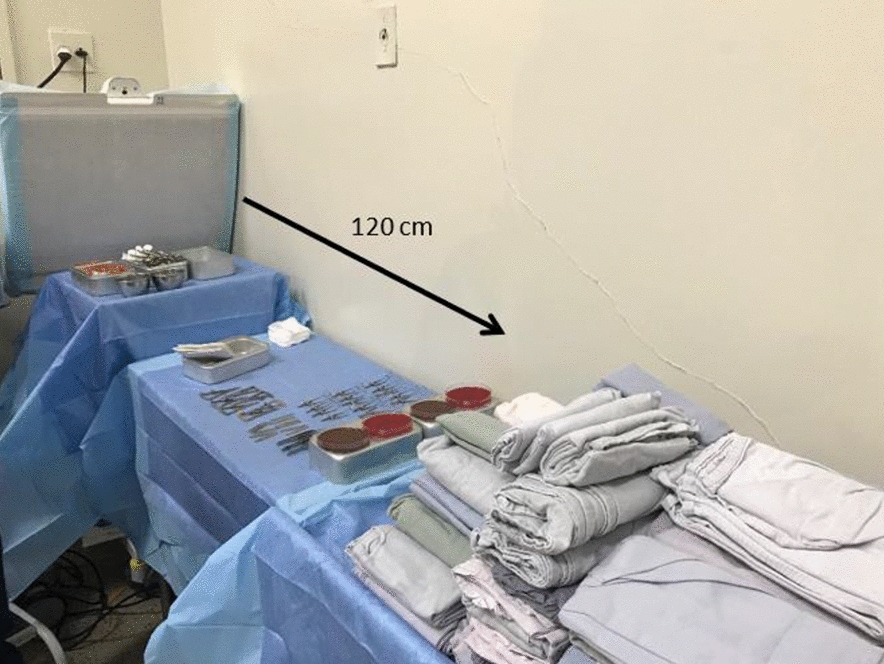
Surgical field with ultra-clean airflow directed towards the surgical table and towards culture plates positioned at a distance of 120 cm from the flow outlet

The UA was obtained by means of a mobile air filter (Operio Mobile^®^, Toul Meditech) which produces a directed, non-turbulent ultra-clean airflow over the sterile instruments. According to the manufacturer’s specifications, this system uses a HEPA filter that eliminates particles smaller than 0.3 µm from the air. The air flow speed is 0.4–0.5 m/s, with a capacity to clean the air of 400 m^3^/h and a protection area of up to 120 cm (Figs. 1 and 2).

The culture plates were covered while still on the surgical field and promptly taken to the Microbiology Laboratory of the University Hospital, School of Medicine of Ribeirão Preto. After 24 h of incubation at 37 °C, the plates were subjected to a biomolecular study of microorganisms in BioMerieux^®^ equipment. This equipment identifies which species of microorganism grew on blood Agar and chocolate Agar.

## Results

All culture plates showed microorganism growth, which are described in Table 1.

**Table 1:** Microorganisms identified in culture medium positioned on the surgical table of intravitreous injections at the University Hospital, Faculty of Medicine of Ribeirão Preto with and without receiving ultraclean airflow directed at the surgical field

Microorganism identified in culture medium positioned on the surgical field and receiving ultraclean airflow	Microorganisms identified in culture medium positioned on the surgical field without receiving ultraclean airflow
*Micrococcus* sp	*Staphylococcus haemolyticus* *Staphylococcus aureus* *Staphylococcus cohnii* ssp *urealyticus* *Bacillus* sp

**Fig. 3:**
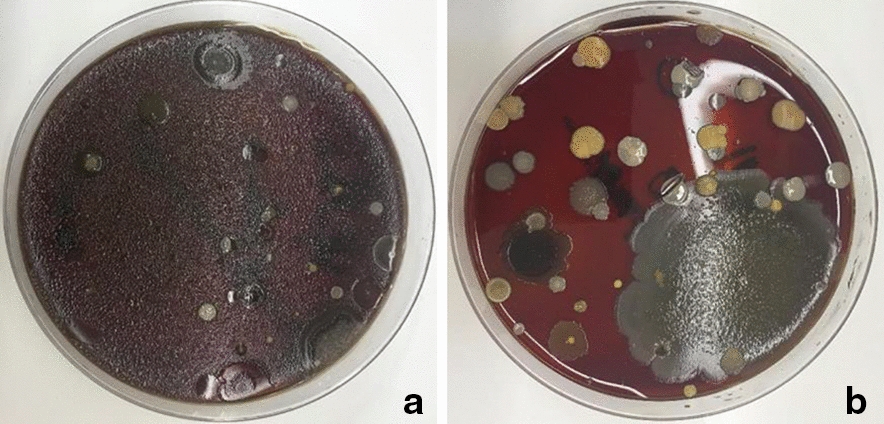
Images of culture medium plates provided by the Microbiology Laboratory of HCFMRP-USP after incubation at 37 °C for 24 h. Plate **a** blood agar, was positioned on the surgical table with ultraclean airflow directed at it. Plate **b** was positioned on the surgical table without receiving an ultraclean airflow

The biomolecular analysis of the culture plates positioned on the surgical tables during use of the UA revealed the growth of bacteria of the genus *Micrococcus* sspp with a 95% probability of success (Table 1, Fig. 3).

The biomolecular analysis of the culture plates positioned on the surgical tables without the use of the UA revealed the growth of bacteria of the genus *Bacillus* sspp with a 97% probability of success, as well as the growth of the following other species, with their respective probabilities of success: *Staphylococcus haemolyticus* (99%), *Staphylococcus aureus* (98%) and *Staphylococcus cohnii ssp urealyticus* (96%). (Table 1, Fig. 3).

In all four IVIs days, there were no cases of EO, with or without the use of UA.

## Discussion

Antisepsis with iodine-povidone, eyelid retraction with speculum, use of face masks to prevent the spread of droplets by the medical team and patient and reduced speech in OR, are recommended measures that may contribute to the reduction in EO after IVIs [7, 10]. On the other hand, there are studies suggesting that intravitreal injections may be performed in the office setting with iodine-povidone and sterile tip technique only, without the use of sterile gloves and sterile drapes [9]. Despite the controversy of more or less rigorous measures to prevent EO, the present study adds additional information on a new tool that maybe used during intravitreal injections either in the operating room, or in the office setting.

One of the reasons to recommend the performance of IVIs in a surgical environment and not in the office, is the OR air quality and the theory that air borne particles may cause infection. The first report of the efficacy of UA in reducing the rates of surgical infection was in orthopedic implant surgery [15]. In ocular surgeries, ESCRS conducted a post-phacoemulsification EO study comparing minimum airflow, air changes per hour and UA systems using horizontal or vertical laminar airflow systems with no clear results [16]. However, in another study that simulates the IVIs environment, the unidirectional air flow showed protection for both the instrument table and the ocular surface, showing that UA can therefore prevent sufficiently infections in the context of IVIs [17].

From our samples of microorganisms that showed growth in the culture plates, we observed that in the presence of UA, there was growth of *Micrococcus* sspp, a Gram-positive bacteria 0.3 to 3.5 µm in diameter, usually present in the normal flora of the skin, mucosa and oropharynx, and far known to have no virulence mechanisms [18].

Among the microorganisms deposited on the culture plates in the surgical field without the UA, *S. cohnii*, S. *haemolyticus* (both bacteria of the CONS group) and *S. aureus* are known to be etiological agents of OE after intraocular procedures. In addition, *S. cohnii* is related to rare cases of endocarditis, pneumonias, urinary tract infections, brain abscesses, and septic arthritis, among another infections [18–20]. When isolated from human infections, *S. cohnii* shows a multiresistant profile. Finally, *S. haemolyticus* is the second species most frequently isolated species from human blood cultures and is highly resistant to antimicrobial agents [20].

As for the strain of *Bacillus* sspp that grew, also in the absence of UA, the majority is non-pathogenic, but for a complete analysis of the identified bacillus it would be necessary to apply the BAAR method, in order to rule out the possibility of being a pathogenic agent such as *B. tuberculosis* [18].

Regarding the maintenance of asepsis of the surgical field, the literature has emphasized the importance of maintaining it throughout the surgical period [10, 12, 13].

The data presented in this work reveal that the use of UA directed to the surgical field can be a useful auxiliary tool in an attempt to ensure the maintenance of asepsis of instruments and needles to be used in IVIs, as well as ensuring asepsis during manipulation (aspiration of the medications) of IVIs.

Our work has some limitations. The number of colonies for each isolated microorganism was not verified, and for this reason there was just a qualitative analysis, instead of a quantitative analysis with statistics. In addition, due to the reduced physical space, the UA was not placed so its effect would extend over the ocular surface, which is the objective of our next study. Finally, there was not a control group with a device providing airflow that was not “ultraclean”, to check if the absence of pathogenic bacteria growth may be due to rapid air flow and not the HEPA filtered air.

The use of UA close to the operating table prevented the growth of pathogenic bacteria and should be considered as an alternative tool to avoid the contamination of materials and drugs used for intravitreal injections.

## Data Availability

All data generated or analysed during this study are included in this published article [and its supplementary information files].
